# The immune landscape of cerebrospinal fluid across brain malignancies

**DOI:** 10.1093/noajnl/vdag058

**Published:** 2026-02-27

**Authors:** Jee Won Kim, Shih-Ying Wu, Abhishek Tyagi, Kounosuke Watabe

**Affiliations:** Department of Cancer Biology, Wake Forest Baptist Medical Center, Winston-Salem, North Carolina; Department of Cancer Biology, Wake Forest Baptist Medical Center, Winston-Salem, North Carolina; Department of Radiation Oncology, Wake Forest Baptist Medical Center, Winston-Salem, North Carolina; Department of Cancer Biology, Wake Forest Baptist Medical Center, Winston-Salem, North Carolina; Department of Cancer Biology, Wake Forest Baptist Medical Center, Winston-Salem, North Carolina

**Keywords:** brain malignancies, cerebrospinal fluid, immune microenvironment, leptomeningeal metastasis, ­liquid biopsy

## Abstract

Cerebrospinal fluid (CSF) is an active immunological interface within the central nervous system, reflecting and shaping tumor–immune interactions that are often invisible in peripheral blood. Accumulating evidence demonstrates that CSF immune remodeling is highly malignancy-specific rather than uniform across brain cancers. In this review, we synthesize recent advances in CSF immune profiling across gliomas, primary CNS lymphomas, brain metastases, and leptomeningeal metastases, highlighting how each disease programs distinct cellular and cytokine circuits. Gliomas are characterized by myeloid skewing and IL-6–associated inflammation, CNS lymphomas by IL-10/CXCL13-driven B-cell–supportive niches, brain metastases by primary tumor–imprinted inflammatory signatures, and leptomeningeal metastases by profound macrophage dominance and complement-mediated immune reprogramming. Importantly, CSF alterations are not merely correlative but mechanistically linked to immune evasion, disease progression, and therapeutic responsiveness. We further discuss translational applications of CSF analysis for molecular stratification, immune monitoring, and treatment response assessment, and critically evaluate the emerging role of CSF-directed therapeutic strategies. Collectively, this review positions CSF as a biologically and clinically integrated compartment with growing relevance for precision neuro-oncology.

Key Points

- Brain malignancies reshape the CSF immune microenvironment in disease-specific patterns rather than through a uniform immunosuppressive program.
- Each malignancy is characterized by distinct CSF immune programs, including IL-6-associated myeloid inflammation in glioma, IL-10/CXCL13-mediated B cell support in CNS lymphoma, primary tumor-specific inflammatory signatures in brain metastases, and macrophage-dominated reprogramming in leptomeningeal metastases.
- CSF profiling often outperforms plasma for capturing CNS-private molecular and immune features, supporting its use in stratification, monitoring, and therapeutic development.

The central nervous system (CNS) has long been considered an immune-privileged organ, largely due to its unique anatomical barriers including the blood-brain barrier (BBB), blood-cerebrospinal fluid barrier (BCSFB), and the reduced presence of conventional lymphatic drainage.^1^^,^^2^ However, the discovery of meningeal lymphatic vessels and the growing evidence of active immune cell trafficking in and out of the CNS have reshaped this notion into “immune specialization” rather than absolute exclusion.^3^ In parallel, the development of advanced molecular techniques, including single-cell RNA sequencing (scRNA-seq) and liquid biopsy approaches, has enabled researchers to study the CNS immune microenvironment in finer detail.^4^

Among the various components that orchestrate immune regulation in the CNS, the cerebrospinal fluid (CSF) plays a pivotal role. The CSF, encompassing the ventricular system, subarachnoid spaces, and perivascular spaces,^5^ not only provides mechanical protection but also serves as a carrier for immune cells and cytokines.^6-8^ Immune cells detected in the CSF originate from multiple interconnected sources, including peripheral blood, meningeal compartments, and the choroid plexus.^9-11^ Through continuous trafficking within the CSF, these cells survey CNS border regions and contribute to immune communication at the brain-CSF interface.^10^^,^^11^ Therefore, analyzing CSF can offer valuable insights into the immunological and molecular changes associated with brain malignancies. While the CSF directly reflects the tumor microenvironment in leptomeningeal metastases, CSF alterations in brain parenchymal tumors, such as gliomas, CNS lymphomas, and brain metastases, likely represent indirect manifestations shaped by CNS anatomical barriers. Nonetheless, diverse CNS malignancies actively reshape the CSF immune landscape to promote tumor survival and evade immune responses. Each type of malignancy employs distinct yet overlapping mechanisms of immune modulation. These immune remodeling events in the CSF underscore the value of CSF-based liquid biopsy for both diagnosis and disease monitoring.^12^

In this review, we provide a comprehensive overview of the immune cell populations and cytokine profiles in the CSF across various CNS malignancies, highlighting how each tumor type orchestrates its distinct immunosuppressive or pro-inflammatory niche. We also discuss how such insight can lead to the development of more effective immunotherapy strategies that specifically target the intricate immune barriers within the CNS. Through an in-depth understanding of the CSF immune landscape, we aim to illuminate new paths toward precision medicine approaches that integrate diagnostic, prognostic, and therapeutic dimensions tailored to the unique characteristics of individual patient’s CNS tumors.

## CSF as a Window into the Brain

### Function

CSF serves multiple critical functions within CNS. First, it provides mechanical protection by surrounding the brain with a liquid cushion that prevents direct contact with the skull and shields it from physical shocks.^6^^,^^8^^,^^13^ This buoyant effect reduces the brain’s apparent weight by 10–15 times.^8^ Although the human brain weighs approximately 1.2–1.4 kg, it exerts a load equivalent to only about 4% of its mass within the cranial cavity.^6^^,^^8^^,^^13^ CSF also contributes to chemical homeostasis by regulating electrolyte levels and transporting hormones and neurotransmitters necessary for CNS stability and function.^13^^,^^14^ Additionally, CSF supports nutrient transport, delivering essential substances required by the CNS, while facilitating waste removal by eliminating toxins and metabolic byproducts.^13^^,^^14^ Furthermore, CSF functions as a regulated bidirectional transport conduit, supporting molecular and cellular exchange between the CNS and peripheral compartments.^15^^,^^16^ This trafficking encompasses CNS-derived metabolites and antigens as well as circulating immune mediators and cells, including amyloid-β, tau, ­cytokines, chemokines, immunoglobulins, extracellular vesicles, and immune cells.^17-21^ Through these continuous and coordinated exchange, CSF links metabolic clearance with immune surveillance, and provides a composite readout of CNS physiological and pathological states within the CNS.^22^^,^^23^ Because of these properties, CSF has substantial diagnostic and therapeutic importance. It serves as a readily accessible medium for diagnosing CNS diseases and enables drug administration directly to the CSF, which is particularly relevant for the treatment of leptomeningeal metastases, while generally exhibiting limited penetration into the brain parenchyma.^14^^,^^24^

### Generation and Circulation

CSF is primarily produced by choroid plexus, located within the brain’s ventricles.^6^^,^^8^^,^^25-27^ This structure filters blood plasma to form CSF at a rate of 400–600 mL per day in adults, though the total volume within the CNS at any given time is about 150 mL in adults due to continuous circulation and drainage.^6^^,^^8^^,^^25-27^ Approximately 70% of CSF originates from the choroid plexus, with the remaining fraction derived from other sources, such as cerebral capillaries and ependymal cells.^25^ CSF is initially produced by passive plasma ­filtration from choroidal capillaries, followed by active transport across choroidal epithelial cells, where ion pumps and aquaporins regulate its composition and flow.^6^^,^^8^^,^^25-27^ CSF circulation begins in the lateral ventricles, moves through the third and fourth ventricles, and then enters the subarachnoid space, surrounding the brain and spinal cord.^25^^,^^27^ This circulation is driven by arterial pulsations and respiratory movements.^8^^,^^25^ CSF drainage primarily was traditionally thought to occur predominantly through arachnoid granulations into the dural venous system.^6^^,^^25^ However, accumulating evidence from both animal models and human studies indicates that this pathway alone does not account for total CSF outflow. Instead, meningeal lymphatic vessels located within the dura mater play a major role in CSF drainage, facilitating the transport of CSF-derived ­macromolecules to cervical lymph nodes.^28^^,^^29^ Additional clearance pathways include perineural lymphatics and connections to the nasopharyngeal lymphatic plexus, underscoring the close integration between CSF circulation and the peripheral lymphatic system.^29-34^ In addition to classical CSF circulation, the glymphatic system has emerged as a critical mechanism governing CSF-interstitial fluid exchange within the brain parenchyma.^35^ Driven by arterial pulsatility and aquaporin-4-dependent astrocytic pathways, the glymphatic system facilitates the clearance of metabolic waste and immune-relevant molecules.^35^^,^^36^ Dysregulation of glymphatic flow has been implicated in neuroinflammatory and neurodegeneration conditions, further highlighting its relevance to CSF dynamics in both health and disease.^37^

### Cellular Components

Under normal physiological conditions, the cellular content of CSF is minimal and highly regulated to maintain CNS homeostasis. In healthy adults, CSF is largely acellular, ­typically containing 0 to 5 white blood cells (WBCs) per microliter (µl).^7^^,^^38^^,^^39^ The presence of red blood cells (RBCs) in CSF generally indicates a pathological condition, such as ­hemorrhage or a traumatic lumbar puncture, rather than a normal finding.^7^ The cellular composition of CSF is pre­dominantly lymphocytic, with a normal lymphocyte-to-monocyte ratio of approximately 2:1 to 3:1 in adults,^7^ though this balance may shift in infants, where monocytes are more prevalent.^40^ Such low cell counts are crucial for preventing unwanted immune activation in the CNS, as even minor increases can signal inflammation or pathology, including infections,^41^ autoimmune conditions,^42^ or malignancy-associated inflammation^43^^,^^44^ in the CSF space. Despite the low steady-state cellularity of CSF, immune cells present within this compartment are not static but dynamically traffic between peripheral blood, meningeal interfaces, and the choroid plexus.^9-11^^,^^45^ In particular, accumulating evidence indicates that a substantial proportion of CSF immune cells originate from the meninges, including the dura and leptomeninges, which harbor resident and circulating immune populations capable of surveilling CNS-adjacent spaces.^10^^,^^17^^,^^46^ These meningeal immune cells can access the CSF and dynamically redistribute within the CSF space, contributing to immune monitoring along CNS border regions. In parallel, the choroid plexus serves as a specialized immunological gateway, enabling regulated entry of peripheral immune cells into the CSF via the blood–CSF barrier.^11^

Among immune cells, T lymphocytes predominate in normal CSF, with CD4+ T cells comprising the majority.^47^ These cells characterized by prior antigen experience and the capacity for rapid recall responses upon re-stimulation and are thought to play a key role in CNS immune surveillance.^47^ CD4+ T cell counts typically range from 0.08 to 1.43 cells/µl.^38^^,^^47^ Small populations of other cell types including CD8+ T cells (0.04–0.40 cells/µl), which contribute to cytotoxic immune responses, B lymphocytes (0.00–0.03 cells/µl), responsible for antibody production, and natural killer (NK) cells (0.00–0.05 cells/µl), which mediate innate cytotoxicity, are occasionally present but comprise a very minor fraction of the total cellularity.^38^^,^^47^ Dendritic cells (DCs), key antigen-presenting cells, including both myeloid and plasmacytoid types, are detectable at low frequencies (0.01–0.18 cells/µl), suggesting potential roles in CNS immune surveillance, although their specific functions in healthy CSF remain under investigation.^38^^,^^47^ Occasionally, procedural contaminants may introduce non-native cell types into CSF. Chondrocytes and fibroblasts may appear following lumbar puncture, while small clusters of choroid plexus or ependymal cells are sometimes found in ventricular CSF samples, particularly in young children or individuals with hydrocephalus.^48^ Notably, CSF cellular composition and soluble factor concentrations may vary depending on the site of sampling, with lumbar CSF often differing quantitatively from ventricular or Ommaya reservoir-derived CSF, a consideration that is particularly relevant when interpreting clinical and translational studies.^49-51^

### Acellular Components

In addition to its limited cellular content, normal CSF contains a variety of acellular components that are essential for maintaining CNS homeostasis. The primary acellular components in normal CSF include proteins, glucose, electrolytes, and various soluble molecules.^7^^,^^8^^,^^13^ Total protein levels in normal CSF are typically low, compared to plasma, ranging from 15 to 45 mg/dL in adults.^7^ The main proteins present include albumin, which is derived from plasma, as well as small amounts of immunoglobulins, predominantly IgG.^13^^,^^52^ The low concentration of immunoglobulins in the CSF may partly reflect limited immunological activity within the CNS. However, it is also strongly influenced by the restrictive nature of CNS barriers. In this context, CSF IgG can arise from limited plasma extravasation and local immunoglobulin production at meninges, rather than indicating an absence of immune processes.^17^ Other proteins such as beta-trace protein and beta-2 microglobulin serve as markers for CSF origin and turnover, respectively.^53^ CSF glucose levels are generally two-thirds that of blood glucose levels, ranging between 50 and 80 mg/dL.^8^^,^^52^ While elevated CSF glucose levels generally have minimal diagnostic importance, low CSF glucose levels may signal conditions like bacterial or fungal infections, as pathogens often consume glucose.^54^^,^^55^ Key electrolytes such as sodium, potassium, calcium, chloride, and magnesium are closely regulated in CSF, maintaining osmotic balance and neuronal excitability.^8^ Sodium and chloride concentrations are similar to plasma levels, whereas potassium is lower in CSF to help control neuronal action potentials.^39^

Normal CSF also contains small amounts of neurotransmitters, cytokines, and metabolic byproducts. Neuro­transmitters like glutamate, gamma-aminobutyric acid (GABA), and dopamine are present in trace amounts,^56^ while cytokines are found at very low levels, reflecting tightly regulated immune activity within the CNS under physiological conditions.^2^^,^^57^ Lactate levels in normal CSF are generally below 3 mmol/L, but they can increase under conditions of CNS hypoxia or inflammation.^58^^,^^59^ Although lipids are present in minimal amounts, they contribute to cellular membrane integrity and neuron function.^60^^,^^61^ Abnormal increases in CSF lipid content may be associated with myelin breakdown, as seen in demyelinating diseases.^62^^,^^63^

## Glioma: Reshaping the CNS Immune Milieu

Gliomas are primary brain tumors derived from glial cells, which play crucial roles in supporting and insulating neurons in the CNS.^64^^,^^65^ Gliomas are highly heterogeneous, and include various subtypes such as astrocytomas, oligodendrogliomas, and glioblastomas, with glioblastoma being the most aggressive.^66^ A hallmark of gliomas is their ability to reshape the immune environment of the CNS, thereby suppressing immune surveillance and facilitating tumor survival, invasion, and progression.^67^^,^^68^ This immune remodeling is not a passive byproduct but an active mechanism through which gliomas establish a supportive microenvironment.

### Immune Cell Populations in Glioma CSF

**Figure 1.**
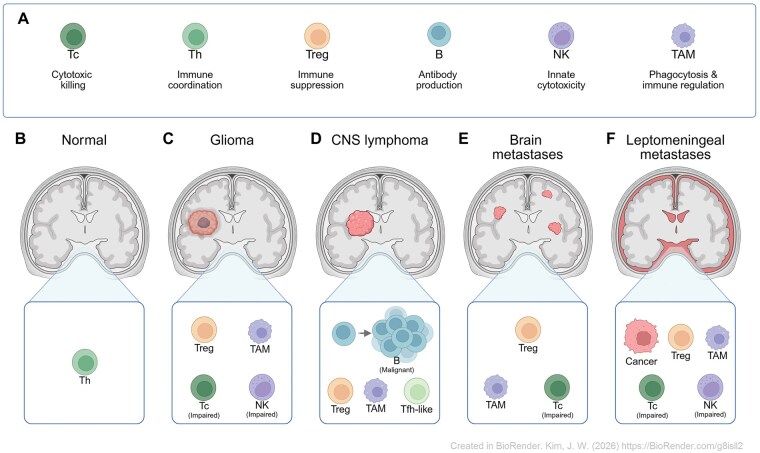
Altered immune cell populations in cerebrospinal fluid of brain malignancies. (A) Canonical immune functions of major immune cell populations detected in the cerebrospinal fluid (CSF), including cytotoxic T cell (Tc cell), helper T cell (Th cell), regulatory T cell (Treg cell), B cell, natural killer cell (NK cell), and macrophage (tumor-associated macrophage, TAM). (B) Normal CSF contains a minimal and tightly regulated immune cell repertoire, dominated by Th cells. (C) In glioma, CSF shows enrichment of Tregs and TAMs, accompanied by impaired T and NK cell function. (D) In primary CNS lymphoma (PCNSL), malignant B cells predominate in the CSF, together with Tregs, supportive myeloid populations, and T follicular helper–like (Tfh-like) cells. (E) In parenchymal brain metastases (BrM), CSF exhibits a myeloid-skewed immune landscape with impaired T and NK cell compartments. (F) In leptomeningeal metastases (LM), tumor cells directly occupy the CSF space, with pronounced myeloid dominance, Treg enrichment, and impaired T/NK cell activity. Figure created with BioRender.com.

In contrast to the relatively immune-stable environment of the healthy CNS, glioma is associated with prominent remodeling of the CSF immune compartment (Figure 1 and Table 1). Single-cell profiling of CSF from patients with glioblastoma consistently has shown a myeloid-skewed landscape, with significant enrichment of macrophages.^69^^,^^70^ The myeloid predominance is further supported by elevated levels of tumor-associated macrophages (TAMs)-related markers, including CD163, as well as macrophage-associated cytokine signatures that correlate with disease aggressiveness and clinical prognosis in high-grade ­glioma.^70^^,^^71^ Within this myeloid-dominant milieu, regulatory T (Treg) cells are frequently increased in glioma CSF, consistent with an inhibitory immunological environment.^69^ In parallel, flow-cytometric profiling of intrathecal lymphocytes in IDH-wildtype glioma demonstrates altered adaptive immunity characterized by signatures of T cell exhaustion, with increased expression of exhaustion markers also detectable on NK-cell populations.^77^ Collectively, glioma CSF exhibits a convergent immune phenotype characterized by enrichment of tumor-associated myeloid cells, Treg expansion, and dysfunction of Tc and NK cell compartments.

**Table 1.** Increased immune cell types in human csf of brain malignancies

Immune cell type	Malignancy	Functional role in CSF/tumor microenvironment	References
Tumor-associated ­macrophage (TAM)	Glioma, PCNSL, BrM, LM	Immunosuppressive polarization (IL-10, TGF-β); promote angiogenesis, invasion, extracellular matrix remodeling; major myeloid dominance in LM	^44^ ^,^ ^70-72^ ^,^ ^73^ ^,^ ^74^ ^,^ ^75^ ^,^ ^76^
Regulatory T cell (Treg)	Glioma, PCNSL, BrM, LM	Enriched across multiple malignancies; suppress antigen presentation and effector activity; expansion linked to IL-10/TGF-β signaling	^44^ ^,^ ^70^ ^,^ ^73^ ^,^ ^74^ ^,^ ^77^
Cytotoxic T cell (CD8^+^ Tc)	Glioma, PCNSL, BrM, LM	Present but functionally exhausted/dysfunctional; reduced clonal expansion; fail to mount effective cytotoxic responses	^44^ ^,^ ^78^ ^,^ ^73^ ^,^ ^74^ ^,^ ^77^
Natural killer cell (NK)	Glioma, LM	Reduced effector activity; exhausted phenotype; in LM, NK activation can be restored by CCR7^+^ DC circuit	^78^ ^,^ ^77^
T follicular helper-like cells (Tfh-like, CXCR5^+^PD-1^+^CD4^+^)	PCNSL	Promotes B cell proliferation and survival via CD40L and IL-21	^79^

Abbreviations: BrM, brain metastases; LM, leptomeningeal metastases; PCNSL, primary CNS lymphoma.

### Cytokine Profiles in Glioma CSF

The cytokine milieu of glioma CSF closely aligns with the myeloid-enriched immune landscape observed in this compartment (Table 2). Among the most consistently detected alterations, interleukin (IL)-6 is elevated in the CSF of glioma patients and has been associated with adverse clinical outcomes.^71^ Notably, CSF IL-6 concentration correlates with tumor-associated macrophage infiltration, supporting a biological link between myeloid activation and the CSF cytokine remodeling.^71^ Angiogenesis-related signal is also a prominent feature of glioma CSF. Vascular endothelial growth factor (VEGF) levels and angiogenic capacity in CSF have been associated with glioma grade and prognosis, suggesting that CSF proteins may capture clinically relevant vascular remodeling programs.^94-96^ Beyond these factors, alterations in homeostatic and immunomodulatory cytokines further reflect immune dysregulation within the CSF compartment. Reduced CSF IL-7 levels in glioblastoma patients have been reported and are consistent with impaired T cell homeostasis.^97^ In addition, intratumoral macrophage infiltration has been shown to correlate with multiple CSF cytokines, including eotaxin, IFN-γ, IL-1β, IL-2, IL-10, IL-13, IL-16, and VEGF, highlighting coordinated crosstalk between the glioma microenvironment and the CSF immune milieu.^97^ Collectively, current CSF-focused studies most consistently support an IL-6 and VEGF-associated cytokine profile in glioma, consistent with myeloid-driven inflammation and vascular remodeling, while broader immunosuppressive cytokine claims should be grounded in CSF-specific primary evidence when available.

**Table 2.** Increased cytokines/chemokines in human CSF of brain malignancies

Cytokine/chemokine	Malignancy	Functional role in CSF/tumor microenvironment	References
Complement component 3 (C3)	LM	Reprograms CSF immune responses; recruits/polarizes macrophages; paradoxical inflammation that benefits tumor growth	^80^
CXCL10	BrM	IFN-inducible chemokines recruiting CXCR3+ T cells; infiltration occurs but effector function is limited	^81^ ^,^ ^82^
CXCL13	PCNSL	Recruits CXCR5+ B cells and Tfh-like cells; sustains intrathecal B-cell proliferation	^83^ ^,^ ^84^ ^,^ ^85^
IFN-γ	BrM, LM	In LM: supports protective CCR7+ DC–NK cytotoxic circuit	^81^ ^,^ ^86^
IL-1β	LM	Reflects myeloid activation and endothelial dysfunction; correlates with radiographic progression	^87^ ^,^ ^88^
IL-6	Glioma, LM	Activates STAT3 signaling, driving proliferation, invasion, and angiogenesis; correlates with symptom burden in LM; predictive marker in BrM immunotherapy	^72^ ^,^ ^87^ ^,^ ^88^
IL-8	BrM, LM	Inflammation-related mediator; promotes myeloid activation, BBB permeability, and tumor progression; rises with LM flare	^87-89^
IL-10	PCNSL, LM	Potent immunosuppressive cytokine; inhibits T-cell activation and antigen presentation; elevated in PCNSL (>50 pg/mL) as diagnostic marker	^83^ ^,^ ^90^ ^,^ ^91^ ^,^ ^88^
IL-17	BrM	Promotes Th17 polarization, neutrophil recruitment, BBB disruption, and glial activation facilitating CNS colonization	^92^ ^,^ ^93^
Lipocalin-2 (LCN2)	BrM, LM	Iron scavenging protein; remodels brain vasculature and supports metastatic colonization	^94^ ^,^ ^87^
TGF-β	LM	Enforces immunosuppression; promotes Treg induction and T-cell anergy; contributes to invasion	^88^
VEGF	Glioma	Enhances angiogenesis and vascular remodeling; associated with poor survival	^95-97^

Abbreviations: BrM, brain metastases; LM, leptomeningeal metastases; PCNSL, primary CNS lymphoma.

**Table 3.** Clinical trials of CSF-directed drug delivery

NCT ID	Title	Malignancy	Intervention	Phase/N	Primary	Secondary	Key stats	Response
NCT01645839^149^	Interest of Intrathecal Chemotherapy With Liposomal Cytarabine (DepoCyte^®^) in Meningeal Metastasis of Breast Cancer	Breast cancer with LM	IT liposomal cytarabine + systemic tx vs systemic tx	Phase 3 /74	LM-PFS	OS, toxicity	LM-PFS 3.8 vs 2.2 mo (HR 0.61, *P* = .04); OS 7.3 vs 4.0 mo	Improved LM-PFS
NCT01325207^150^	Intrathecal Trastuzumab for Leptomeningeal Metastases in HER2+ Breast Cancer	HER2+ breast cancer with LM	IT trastuzumab	Phase 1–2 /34	Safety, RP2D	LM response, OS	Median OS 8.3 mo (HER2 + 10.5 mo)	PR 19%, DCR ∼70%
NCT03507244^152^	Concurrent Intrathecal-pemetrexed and Involved-field Radiotherapy for Leptomeningeal Metastasis From Solid Tumors	Solid tumors with LM	IT pemetrexed + involved-field RT	Phase 1–2 /34	Safety	LM response	Feasible combination; manageable toxicity	NR
NCT05289908^151^	Intrathecal Pemetrexed for Leptomeningeal Metastasis	Solid tumors with LM	IT pemetrexed	Phase 1–2 /34	MTD, safety	OS	Median OS reported; tolerable toxicity	NR
NCT03101579^153^	Intrathecal Pemetrexed for Recurrent Leptomeningeal Metastases From Non-small Cell Lung Cancer	NSCLC with refractory LM	IT pemetrexed	Phase 1 /13	Safety	LM response	Severe AEs 31%	Response 31%, DCR 54%

Abbreviations: AE, adverse event; DCR, disease control rate; HER2, human epidermal growth factor receptor 2; HR, hazard ratio; IT, intrathecal; LM, leptomeningeal metastasis; LM-PFS, leptomeningeal progression-free survival; MTD, maximum tolerated dose; NR, not reported; NSCLC, non-small cell lung cancer; OS, overall survival; PR, partial response; RP2D, recommended phase 2 dose; RT, radiotherapy.

### Therapeutic Implications of CSF Analysis in Glioma

Gliomas display marked spatial and temporal heterogeneity, and repeated tissue biopsies are limited.^98^ In this regard, CSF provides repeatable access to peritumoral biology, enabling integrated readouts of tumor nucleic acids, immune cell states, and cytokine programs. While CSF analysis is not currently used to guide routine treatment decisions based on immune profiling, it represents a valuable investigational window into tumor-immune dynamics that are otherwise difficult to capture longitudinally.

Recent studies demonstrate that CSF liquid biopsy outperforms plasma in detecting glioma-specific alterations, including MGMT promotor methylation and targetable mutations in IDH1/2 and TERT promoter.^99-101^ Beyond genomics, cytokine profiling in CSF has demonstrated prognostic relevance and may inform biologically actionable therapeutic hypotheses. Elevated CSF IL-6 levels and the IL-6/IL-10 ratio have been associated with adverse clinical outcomes in glioma patients, supporting their utility as potential prognostic markers.^71^ Importantly, accumulating preclinical evidence indicates that IL-6-driven signaling is not merely correlative but therapeutically actionable. For example, dual targeting of IL-6 signaling and CD40 produced synergistic anti-tumor immune responses and survival benefit in glioblastoma models, establishing IL-6/STAT3-associated pathways as viable targets for immunomodulatory intervention.^102^ In this context, CSF analysis may serve as a translational research bridge by linking cytokine-defined immune states to therapeutic vulnerability. Notably, emerging evidence indicates that the CSF constitutes an active functional context that can modulate therapeutic responsiveness rather than serving solely as a source of correlative biomarkers.^103^ Exposure of patient-derived glioblastoma cells to autologous CSF has been shown to modulate sensitivity to chemoradiotherapy, indicating that CSF-borne factors functionally influence treatment response and reinforcing the relevance of CSF analysis as a mechanistic and therapeutic context.

Microdialysis-based frameworks demonstrate the feasibility of real-time local immune monitoring during checkpoint blockade, suggesting that serial CSF sampling could serve as an early pharmacodynamic readout, although such approaches remain investigational and not yet clinically validated.^104^ Looking forward, integrating CSF cytokine signatures with CSF-based mutation tracking may enable biologically informed patient stratification and adaptive trial designs, including evaluation of strategies targeting IL-6/STAT3 signaling, macrophage polarization, or angiogenic pathways such as VEGF. Thus, while CSF immune profiling does not currently guide routine treatment decisions, it represents a mechanistically informative platform with clear potential to support precision immunotherapy development in glioma.

## CNS Lymphoma: B-Cell Malignancies and Immune Dysregulation

Primary CNS lymphomas (PCNSL) is an aggressive subtype of diffuse large B-cell lymphoma confined to the brain, spinal cord, eyes, and leptomeninges without systemic involvement at diagnosis.^105^ By adapting to the unique immune environment of the CNS, PCNSL reprograms perivascular and meningeal microenvironments, blunts cytotoxic surveillance, and drives invasive behavior and high relapse risk despite initial chemosensitivity.^106^^,^^107^ In this regard, PCNSL provides a model of how malignant B cells achieve long-term persistence by circumventing CNS immune surveillance mechanisms.

### Immune Cell Populations in CSF of CNS Lymphoma

Leptomeningeal or CSF involvement of PCNSL is detected in a subset of patients, with reported frequencies ranging from approximately 7-42%, depending on diagnostic modality and criteria.^108^ Primary leptomeningeal PCNSL without parenchymal disease is rare, and most cases of CSF involvement arise in the context of parenchymal lymphoma.^109^ Importantly, the relevance of CSF analysis extends beyond overt leptomeningeal disease. Neuroimaging studies show that approximately 97% of parenchymal PCNSL lesions directly contact CSF surfaces, highlighting the close anatomical relationship between tumor cells and the CSF compartment.^110^ This near-universal proximity provides a strong biological rationale for CSF-based interrogation of tumor and immune features, even in the absence of cytologically detectable CSF dissemination.

When present, CSF involvement is characterized by the detection of malignant or clonally expanded B cells, which are thought to originate from tumor populations occupying perivascular and meningeal interfaces and to access the CSF via Virchow-Robin spaces.^44^^,^^111^ Beyond the malignant component, the CSF immune landscape typically shows and effector-low, regulatory- and myeloid-skewed state (Figure 1 and Table 1). While Treg cells and TAM-like populations are relatively enriched, Tc cells are present but exhibit exhaustion and attenuated cytotoxic programs, mirroring the intratumoral microenvironment and correlating with unfavorable clinical outcomes.^72^^,^^73^ In addition to these canonical shifts, the enrichment of CXCR5^+^PD-1^+^CD4 T follicular helper-like (Tfh-like) cells is distinguished in PCNSL CSF.^78^ These cells are known to support B cell proliferation and survival through CD40L and IL-21 signaling, promoting germinal-center programs such as BCL6.^112^^,^^113^ In the context of PCNSL, the presence of Tfh-like cells may therefore contribute to malignant B cell persistence and immune evasion, suggesting that the tumor leverages adaptive immune components to sustain tumor survival.

### Cytokine Profiles in CSF of CNS Lymphoma

Cytokine signatures in PCNSL CSF further underscore its immunosuppressive environment (Table 2). Among these, IL-10 is the most consistently elevated cytokine, often exceeding 50 pg/mL, compared to undetectable levels in normal CSF.^82^^,^^89^^,^^90^ Functionally, IL-10 is a potent immunoregulatory cytokine that suppresses T cell activation and antigen presentation, thereby attenuating anti-tumor immune responses.^114^ Beyond its immuno-modulatory effects, IL-10 signaling in B cell lymphoma has been linked to activation of JAK/STAT3 pathways and enhanced tumor cell survival.^115^ The marked elevation of IL-10 in PCNSL CSF therefore suggests that this cytokine may contribute not only on immune suppression but also to the persistence of malignant B cells within the intrathecal compartment. CXCL13 is another defining cytokine in PCNSL CSF.^82-84^ As a key B cell chemoattractant, it contributes to the recruitment and retention of CXCR5^+^ B cells and Tfh-like cells.^112^^,^^113^ Elevated CSF CXCL13 levels have been associated with active disease states and tend to decrease following treatment response.^84^^,^^116^^,^^117^ Collectively, these cytokine patterns reflect a CSF environment characterized by immunoregulation mediated by IL-10 and by chemokine-dependent recruitment of B cells, both contributing to the maintenance and progression of CNS lymphoma.

### Therapeutic Implications of CSF Components in CNS Lymphoma

From a translational perspective, CSF analysis has emerged as a powerful diagnostic and monitoring tool in PCNSL. Measurement of IL-10 in CSF markedly improves presurgical discrimination between PCNSL and inflammatory or other malignant conditions.^82^^,^^89^^,^^90^^,^^111^ More importantly, the IL-10/IL-6 ratio in CSF offers superior diagnostic accuracy, with a sensitivity of 95.5% and specificity of 96.1%, and outperforms IL-10 alone.^82^^,^^89^^,^^90^^,^^118^ Beyond diagnosis, CSF cytokines provide actionable markers for treatment monitoring. Serial IL-10 measurement reflects therapeutic response: early declines during high-dose methotrexate-based therapy associated with radiographic/clinical response, whereas persistence or rebound predicts early relapse and shorter progression-free survival.^89^^,^^119^ In this regard, CSF cytokine monitoring could be integrated into adaptive treatment protocols, allowing therapy to be escalated or modified in near-real time. Moreover, CSF profiling may also guide novel therapeutic strategies. For example, IL-10 blockades or modulation of CXCL13/CXCR5 signaling could be explored to disrupt the tumor growth in CSF niche.^89^ Furthermore, as immune checkpoint inhibitors are increasingly considered for PCNSL, baseline CSF cytokine states (eg, IL-10/IL-6 ratio, Tfh-like enrichment) could serve as predictors of response or resistance to the therapy of immune checkpoint inhibitor.^120^^,^^121^ Therefore, incorporating CSF analysis into clinical trial design will be critical, not only for patient selection but also for pharmacodynamic readouts.

## Brain Metastases: Systemic Tumors Adapting to CNS

Brain metastases (BrM) are secondary tumors that arise when cancer cells from extracranial primary tumors migrate to and colonize the CNS.^122^ These metastases are the most common intracranial tumors in adults, significantly contributing to morbidity and mortality.^123^ Unlike primary CNS tumors, parenchymal BrM must first break CNS barriers and then adapt to a non-native microenvironment.^124^ During this adaptive process, metastatic cells actively modulate local immunity to secure survival and growth within the CNS niche.^125^ Although the CSF does not fully capture these parenchymal changes due to spatial constraints, it still provides a minimally invasive window into the underlying biology.^126^

### Immune Cell Populations in Brain Metastases CSF

Parenchymal BrM are anatomically distinct from CSF spaces, and thus the immune signals detectable in CSF depend on lesion size and proximity.^127^ Therefore, BrM CSF partially mirrors the immune state of the intracranial lesions but it nevertheless provides a clinically meaningful window into intracranial immunity.^43^^,^^128^ Indeed, single-cell profiling and TCR genotyping demonstrate shared clonotypes between BrM tissue and CSF, underscoring that CSF can capture ongoing T cell responses.^43^ At the level of immune activity, BrM CSF exhibits a general immunoregulatory profile, with reduced expression of cytotoxic effector programs and diminished inflammatory transcription signatures compared to matched tumor tissue and non-malignant CSF^43^ (Figure 1 and Table 1). Despite this shared immunoregulatory context, BrM CSF retains immune features that reflect the biology of the primary tumor. For example, CSF of melanoma-derived BrM shows substantial T cell infiltration but with limited effector performance.^76^ Furthermore, in breast cancer BrM, CSF is characteristically myeloid-heavy, with prominent monocyte/TAM signatures and variable neutrophil involvement.^74^ These patterns suggest that although immune signals in BrM CSF may be attenuated relative to tumor tissue, they still capture tumor-associated shifts in immune cell composition and functional polarization within the CNS.

### Cytokine Profiles in Brain Metastases CSF

CSF cytokine profiles in BrM depart from the IL-10-enriched immunosuppression which is typical of PCNSL, and are dominated by IFN/chemokine-driven signals and inflammation-associated mediators^80^^,^^91^ (Table 2). In NSCLC BrM CSF, IFN-inducible CXCL10 and CXCL11 are elevated, which are known to recruit CXCR3^+^ T cell.^43^^,^^129^ Also, elevated IL-17-associated inflammatory signaling has been reported in NSCLC BrM CSF,^91^^,^^92^ which has been implicated in neutrophil recruitment, BBB disruption, and glial activation that facilitate brain colonization of cancer cell, based on established mechanistic studies.^130^ In melanoma BrM, CSF exhibits a shift toward CXCL10/CCL4/CCL17/IL-8 with reductions in IL-1α/IL-4/IL-5/CCL22, which is consistent with a T cell recruiting yet function-limited inflammation that supports tumor persistence.^80^^,^^81^ Beyond tumor-specific cytokine ­signatures, recent translational studies have identified lipocalin-2 (LCN2) as a shared mediator across multiple BrM contexts.^93^^,^^131^^,^^132^ LCN2 is a secreted protein produced by tumor cells and reactive components of the brain microenvironment, including astrocytes and myeloid populations, rather than a CSF-specific factor.^93^ In experimental models of melanoma- and breast cancer–derived brain metastases, LCN2 has been detected in the cerebrospinal fluid and shown to correlate with metastatic burden, suggesting that CSF LCN2 levels may reflect pathological remodeling at the tumor–brain interface.^93^ While these findings support the relevance of LCN2 to CNS metastatic biology, further validation in human CSF cohorts will be required to establish its utility as a CSF-based biomarker.

### Therapeutic Implications of CSF Components in Brain Metastases

For parenchymal BrM, detection of cancer cells in CSF is feasible although it depends on the location of tumor. As the yields of CSF biopsy increase with the size and location of tumors, these features can be used to build a regression model that predicts the patient outcomes.^133^ Interestingly, ctDNA of CSF more accurately captures brain tumor private drivers and resistance mutations—genetic alterations confined to intracranial lesions such as EGFR, ALK, HER2 or BRAF alterations—than plasma ctDNA.^127^^,^^134-136^ In addition to genomics, cytokine profiling also provides prognostic and pharmacodynamic value. In NSCLC BrM, multiplex CSF cytokine profiling has been shown to predict intracranial response to PD-1–based immunotherapy, with higher baseline CSF LAMP3 distinguishing responders, while selected inflammatory cytokines (eg, CXCL10 and IL-18) were lower at baseline and further decreased during treatment.^137^ These findings highlight the value of CSF immune profiling as a non-invasive approach to capture CNS-specific immune states that are not reflected by tumor tissue–based biomarkers. In melanoma BrM, elevated CXCL10/IL-6/IL-8/CCL2 in CSF can provide early readouts of inflammatory rewiring or dissemination risk during systemic and radiation ­therapies.^81^^,^^91^

## Leptomeningeal Metastases: Direct Colonization of Cancer Cells in the CSF

Leptomeningeal metastases (LM) occur when cancer cells infiltrate the CSF and leptomeningeal membranes, creating a unique tumor microenvironment that supports cancer cell survival and proliferation.^138^ LM is associated with advanced cancers such as breast cancer, lung cancer, and melanoma.^139^^,^^140^ The CSF, which serves as the immediate ­environment for LM, undergoes significant immunological and molecular changes that both reflect and contribute to disease progression.^141^^,^^142^

### Immune Cell Populations in CSF of Leptomeningeal Metastases

LM reconfigures CSF into a myeloid-dominant, immunosuppressed niche^76^^,^^138^ (Figure 1 and Table 1). The most consistent cellular shift is the expansion of TAM-like macrophages (CD163^+^/CD206^+^, low HLA-DR), accompanied by monocytic and granulocytic MDSC subsets.^75^^,^^93^ Dural macrophages infiltration across the CSF barrier further increases in myeloid dominance within the leptomeningeal niche.^143^ They can enter the CSF via an SPP1-MMP14 program, acquiring immunosuppressive phenotypes that tightly correlated with LM burden. This dominance of myeloid subsets is compounded by Treg enrichment and dysfunction of T and NK cells, effectively silencing adaptive immune surveillance.^76^^,^^93^ On the contrary, CCR7^+^ migratory DCs enter the CSF, where they activate NK cells and partially restore cytotoxic ­activity.^85^ Thus, LM represents a “tug-of-war” in which tumor-promoting myeloid circuits typically overwhelm antitumor DC/NK interactions, explaining the rapid progression and poor prognosis of LM.

### Cytokine Profiles in Leptomeningeal Metastases CSF

The LM cytokine milieu of LM reflects this duality^80^ (Table 2). On one hand, pro-inflammatory cytokines such as IL-6, IL-8, and IL-1β are consistently elevated, indicating robust myeloid activation, endothelial/BBB permeability, and symptom burden.^80^^,^^86-88^ On the other hand, immunosuppressive mediators including TGF-β and IL-10 are also frequently increased, enforcing T cell anergy, reduced antigen presentation, and Tregs expansion.^87^ A particularly notable factor is complement component 3 (C3), which reprograms CSF immune immunity to favor tumor progression by recruiting/­polarizing macrophages and amplifying paradoxical inflammation that benefits the tumor growth.^79^ A countervailing, LM-specific protective circuit involves IFN-γ-driven CCR7^+^ DCs that activate NK cells within the CSF.^85^ When this axis is intact, cytokine patterns shift toward increased IFN signaling and cytotoxic readouts. Importantly, primary-specific imprints are detectable. CFS of Melanoma LM often shows complement/inflammation-associated activation.^87^^,^^128^ Moreover, breast cancer LM highlights metabolic adaptation signals, including LCN2, which facilitates iron scavenging.^86^ These results underscore that cytokine landscapes of CSF in LM are not uniform but reflect the primary tumor’s biology layered onto the CSF niche.

### Therapeutic Implications of CSF Analysis in Leptomeningeal Metastases

As LM resides directly within the subarachnoid space, CSF analysis provides an unusually direct and actionable platform compared to other CNS malignancies. Circulating tumor cell (CTC) assays for CSF-derived ctDNA have shown superior sensitivity to conventional cytology and outperform plasma in detecting CNS-specific drivers and resistance mutations.^133-135^ These capabilities enable targeted therapy, such as EGFR/ALK inhibitors in NSCLC, HER2-directed regimens in breast cancer, and BRAF/MEK inhibitors in melanoma.^133-135^ Furthermore, CSF immuno-profiling adds clinically relevant value. Levels of IL-6, IL-8, and IL-1β typically rise during radiographic or clinical progression, and longitudinal CSF profiling suggests that inflammatory cytokine signals may decrease with disease control, providing a potential dynamic readout of therapeutic response.^86^^,^^144^^,^^145^ Consistent with this concept, a recent multimodal analysis integrating CSF circulating tumor cell detection, single-cell transcriptomics, and proteomic profiling in breast cancer–derived LM have demonstrated that dynamic changes in tumor burden and inflammatory signaling closely parallel treatment response, underscoring the utility of CSF as a real-time monitoring compartment.^146^ Preclinical evidence further suggests that immune axes identified in LM CSF can be exploited as therapeutic targets. In breast cancer-derived LM mouse models, complement C3 blockade has been shown to reverse choroid-plexus conditioning and limit LM growth.^79^ In melanoma LM mouse models, amplification of the IFN-γ-CCR7^+^ DCs-NK cells circuit increases intrathecal cytotoxicity and reduces tumor burden.^85^ In lung cancer LM mouse models, interruption of dura-derived macrophage trafficking has also been shown to reduce myeloid accumulation and prolong survival.^143^ Together, these findings establish CSF not merely as a diagnostic fluid but as a mechanistic readout of tumor-immune interactions and a potential therapeutic route itself.

## Discussion and Future Prospectus

CSF provides a uniquely direct and repeatable window into intracranial tumor-immune dynamics, capturing features that peripheral blood often misses. This review highlights consistent patterns across CNS malignancies (Figure 1 and Table 1): gliomas create a myeloid/Treg-skewed and effector-poor profile^69^^,^^128^; PCNSL is dominated by malignant B cell with strong IL-10/CXCL13 signals^89^^,^^147^; parenchymal BrM reflect primary-specific inflammatory cytokines and injury reponses^128^; and LM exhibit profound myeloid dominance with T/NK dysfunction.^85^^,^^93^ Importantly, soluble factors within CSF are not passive reflections but appear mechanistically linked to immune remodeling, including IL-10 in PCNSL,^89^^,^^128^ CXCL10/IL-6/IL-8 in BrM,^80^^,^^91^ and complement C3/IL-6/IL-8/IL-1β in LM ^79^^,^^80^^,^^144^ (Table 2).

**Figure 2.**
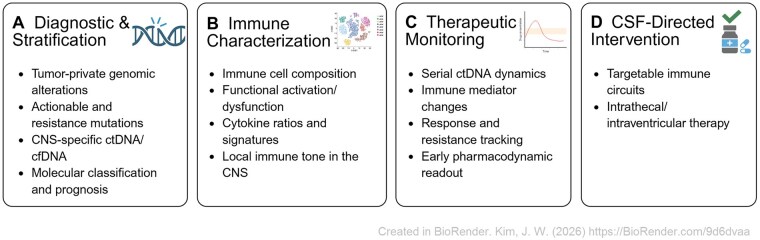
Clinical applications of CSF analysis in brain malignancies. CSF analysis provides a repeatable window into intracranial tumor biology and immune dynamics that are incompletely reflected by peripheral blood. (A) Diagnostic and molecular stratification. CSF-based liquid biopsy enables detection of tumor-private genomic alterations, including actionable and resistance-associated mutations. In selected settings, CSF derived ctDNA and cfDNA more accurately capture CNS-specific molecular features than plasma, supporting molecular classification and prognostic assessment. (B) Immune profiling and disease characterization. Immune profiling of CSF, incorporating immune cell composition and cytokine programs, provides insight into local immune tone and disease activity within the CNS. Distinct immune and cytokine signatures reflect tumor-associated immune remodeling. (C) Treatment monitoring and pharmacodynamic assessment. Serial CSF analysis supports longitudinal monitoring of disease evolution by capturing dynamic changes in tumor-derived nucleic acids and immune mediators. These changes may be associated with therapeutic response, emerging resistance or immune modulation during treatment. (D) Therapeutic development and CSF-directed intervention. Beyond its diagnostic and monitoring roles, CSF represents a biologically active interface where tumor cells, immune populations, and soluble mediators interact. CSF analysis informs the development and evaluation of CSF-directed therapeutic strategies, particularly in leptomeningeal metastases, and supports integration of local interventions with systemic or immunomodulatory therapies. Figure created with BioRender.com

Beyond cellular and cytokine profiling, tumor-derived nucleic acids in CSF (ctDNA/cfNA) frequently outperform plasma for detecting CNS-private mutations and resistance mechanisms, underscoring CSF as a privileged source of molecular interrogation in neuro-oncology^133-135^ (Figure 2). These insights already inform current clinical practice–for example, MGMT methylation and IDH1/2 detection in glioma,^99-101^^,^^104^ IL-10/IL-6 ratios in PCNSL,^89^^,^^118^^,^^119^ PD-L1 response signatures in BrM,^128^^,^^135^^,^^136^ and ctDNA burden in LM^133^ (Figure 2). Collectively, these findings support the concept that CSF analysis can evolve from a diagnostic adjunct toward a dynamic biomarker compartment capable of informing treatment selection and longitudinal monitoring. In this context, serial CSF profiling may function as an immune and molecular readout of intracranial disease activity, particularly in patients receiving immunomodulatory therapies. When integrated with imaging and clinical assessment, CSF-derived biomarkers have the potential to guide adaptive treatment strategies, although this approach remains investigational and requires prospective validation.

Parallel to its diagnostic and monitoring roles, CSF has also been explored as a therapeutic route, particularly in the setting of leptomeningeal metastases (Figure 2). However, clinical outcomes accumulated over the past two decades indicates that CSF-directed therapy occupies a nuanced and selective role rather than a broadly applicable treatment paradigm. Outcomes have varied substantially across tumor types, study designs, and patient populations, reflecting both biological heterogeneity and practical constraints inherent to leptomeningeal disease.

Among prospective studies (Table 3), the randomized DEPOSEIN trial (NCT01645839) provides the clearest signal that sustained drug exposure within the CSF can influence leptomeningeal disease kinetics. In this study, the addition of intrathecal liposomal cytarabine to systemic therapy modestly prolonged leptomeningeal progression-free survival compared with systemic therapy alone, while overall survival benefits remained limited.^148^ Importantly, the investigators emphasized patient selection and clinical context in interpreting these results, underscoring that CSF-directed chemotherapy may be appropriate in specific settings rather than uniformly across all patients with leptomeningeal metastases. Early-phase trials further illustrate both ­feasibility and constraint. Intrathecal trastuzumab in HER2-positive breast cancer leptomeningeal metastases (NCT01325207) demonstrated encouraging disease control and survival outcomes relative to historical expectations, supporting the concept that antibody-based therapies can achieve therapeutically relevant exposure within the CSF compartment with acceptable systemic toxicity.^149^ Similarly, intrathecal pemetrexed has been evaluated across ­several phase I/II studies (NCT03101579, NCT03507244, NCT05289908), showing manageable safety profiles and measurable activity in selected patients, particularly in non-small cell lung cancer.^150-152^ Nevertheless, these studies were not designed to define comparative efficacy, and reported outcomes remain heterogeneous.

Across these trials, a recurring theme is that therapeutic benefit is closely linked to the biological and physical ­properties of leptomeningeal disease itself. Impaired CSF circulation, which is frequently observed in advanced leptomeningeal metastases, can limit drug distribution and increase the risk of neurotoxicity, such as chemical arachnoiditis, reinforcing the importance of pre-treatment assessment and careful monitoring.^153^^,^^154^ Collectively, available data suggest that CSF-directed therapy can meaningfully alter local disease dynamics in selected patients, but its impact is neither uniform nor sufficient to supplant systemic approaches.

These clinical observations align with a broader conceptual shift in how the CSF compartment is viewed. Rather than serving solely as a conduit for drug delivery, CSF represents a biologically active interface where tumor cells, immune populations, and soluble mediators interact. As highlighted earlier, this environment is shaped by distinct immune circuits and cytokine networks that are not readily inferred from peripheral blood. Leveraging CSF as a therapeutic space therefore may be most effective when informed by concurrent biomarker profiling, enabling rational integration of local therapies with systemic or immunomodulatory strategies.

The translational relevance of CSF-directed approaches is likely to depend less on the expansion of conventional intrathecal chemotherapy and more on biologically informed interventions. The CSF compartment is increasingly accessible to diverse therapeutic modalities, including small molecules, antibody-based agents, nanoparticle-formulated drugs, and cellular therapies, primarily in the context of leptomeningeal disease and potentially extending to parenchymal brain tumors. In this framework, CSF should be considered an integrated biological compartment—one that links molecular monitoring with targeted intervention—rather than a passive fluid or a standalone treatment route.

## Data Availability

No new data were generated or analyzed in this study.
